# The interaction between ferroptosis and inflammatory signaling pathways

**DOI:** 10.1038/s41419-023-05716-0

**Published:** 2023-03-21

**Authors:** Yue Chen, Ze-Min Fang, Xin Yi, Xiang Wei, Ding-Sheng Jiang

**Affiliations:** 1 Division of Cardiothoracic and Vascular Surgery, Tongji Hospital, Tongji Medical College, Huazhong University of Science and Technology, Wuhan, Hubei China; 2 Department of Cardiology, Renmin Hospital of Wuhan University, Wuhan, Hubei China; 3 Key Laboratory of Organ Transplantation, Ministry of Education; NHC Key Laboratory of Organ Transplantation; Key Laboratory of Organ Transplantation, Chinese Academy of Medical Sciences, Wuhan, Hubei China

**Keywords:** Cell death, Inflammation

## Abstract

Ferroptosis is an iron-dependent regulated cell death driven by excessive lipid peroxidation. Inflammation is one common and effective physiological event that protects against various stimuli to maintain tissue homeostasis. However, the dysregulation of inflammatory responses can cause imbalance of the immune system, cell dysfunction and death. Recent studies have pointed out that activation of inflammation, including the activation of multiple inflammation-related signaling pathways, can lead to ferroptosis. Among the related signal transduction pathways, we focused on five classical inflammatory pathways, namely, the JAK-STAT, NF-κB, inflammasome, cGAS-STING and MAPK signaling pathways, and expounded on their roles in ferroptosis. To date, many agents have shown therapeutic effects on ferroptosis-related diseases by modulating the aforementioned pathways in vivo and in vitro. Moreover, the regulatory effects of these pathways on iron metabolism and lipid peroxidation have been described in detail, contributing to further understanding of the pathophysiological process of ferroptosis. Taken together, targeting these pathways related to inflammation will provide appropriate ways to intervene ferroptosis and diseases.

## Facts

- Ferroptosis is a form of iron-dependent regulated cell death and has two major hallmarks, intracellular iron overload and redox system disorder.
- The activation of inflammatory signaling pathways, including JAK-STAT, NF-κB, inflammasome, cGAS-STING, and MAPK signaling pathways, is closely related to the occurrence of ferroptosis and vice versa.
- Inflammatory response is also an important inducement for iron metabolism disorder and redox system dysfunction.
- Some novel agents targeting inflammation have shown effective intervention in ferroptosis and thereby exert therapeutic effects on various disease models in vivo.

## Open questions

1. How do we maintain the balance between the intervention of ferroptosis through inflammatory signaling pathways and the stabilization of the immune system?
2. Are the connections among multiple inflammatory signaling pathways unchanged during ferroptosis?
3. Recent studies have only reported this phenomenon that inflammatory pathways have great effects on ferroptosis, but the detailed molecular mechanisms are still unclear.

## Introduction

Cell death is a critical biological phenomenon to maintain tissue homeostasis and participates in multiple physiological and pathological processes [1]. Regulated cell death (RCD) is one spontaneous and programmed cell death mode associated with multiple physiological and pathological processes [2]. Among these novel forms of cell death, ferroptosis is iron-dependent cell death driven by lethal lipid peroxidation [3]. Excessive oxidative stress and disruption of redox systems cause the excessive lipid peroxidation, which further leads to increased mitochondrial membrane density, mitochondrial crest fracture, and the loss of plasma membrane integrity, causing ferroptotic cell death [4, 5]. Therefore, the core molecular mechanism of ferroptosis is the imbalance between oxidative damage and antioxidant defense.

Recently, an increasing number of studies have reported that the activation of inflammation-related signaling pathways is closely connected with ferroptosis [6–8]. Studies have indicated that disordered redox biology and increased lipid peroxidation can activate multiple inflammatory cells and pathways, while proinflammatory cytokines, in turn, aggravate intracellular oxidative stress and excessive lipid peroxidation [6]. For instance, during smooth muscle cell ferroptosis, the NF-κB signaling pathway was activated, and meanwhile the release of proinflammatory cytokines TNF, CXCL1, CXCL8, and CSF2 were increased [8]. In turn, the inhibition of ferroptosis by Trolox decreased the release of the proinflammatory cytokines TNF-α, interleukin (IL)-1β and IL-6, and then alleviated liver injury in the choline-deficient, ethionine-supplemented (CDE) diet model [9]. Hence, focusing on the crosstalk between ferroptosis and inflammation helps to illustrate the respective pathophysiological mechanism of each process and might provide novel therapeutic targets for relevant diseases.

In this review, we first summarized the biological mechanisms of ferroptosis in detail and introduced some key molecules and redox systems that are related to ferroptosis. Next, we described the signal transduction pathways involving five important inflammatory signaling pathways, including the Janus kinase-signal transducer and activator of transcription (JAK-STAT), nuclear factor-κB (NF-κB), inflammasome, cyclic GMP-AMP synthase-stimulator of IFN genes (cGAS-STING) and mitogen-activated protein kinase (MAPK) pathways and provided an overview of the relationship between these pathways and ferroptosis. Finally, based on current studies, we highlighted a few issues that need to be clarified and put forward certain considerations for further researches on inflammation and ferroptosis.

## An overview of ferroptosis

As the name indicates, ferroptosis is an iron-dependent form of cell death and iron overload is the typical hallmark that distinguishes ferroptosis from other cell death [10]. As an essential trace element in the body, iron participates in oxygen transport, DNA biosynthesis and ATP generation to maintain cellular homeostasis [11, 12]. Notably, ferrous iron mediates Fenton reaction and contributes to the generation of reactive oxygen species (ROS) [10, 13]. Moreover, excessive oxygen radicals caused by intracellular iron overload further cause the accumulation of lipid peroxidation products, such as PL-hydroperoxide (PLOOH), malonaldehyde (MDA) and 4-hydroxynonenal (4-HNE), which eventually trigger cell damage and ferroptosis [4]. In general, the membrane-bound protein transferrin receptor (TFR) binds to extracellular transferrin (TF) carrying iron to import iron, while ferroportin is the only known iron exporter in mammalian cells [4, 14–16]. In addition, ferritin is an iron-storage protein that can dynamically regulate the content of intracellular iron as needed, whereas the abnormal degradation of ferritin leads to the release of excessive iron and ferroptosis [10]. In summary, the dysfunction of iron metabolism-related molecules disrupts iron homeostasis and contributes to ferroptosis.

**Fig. 1:**
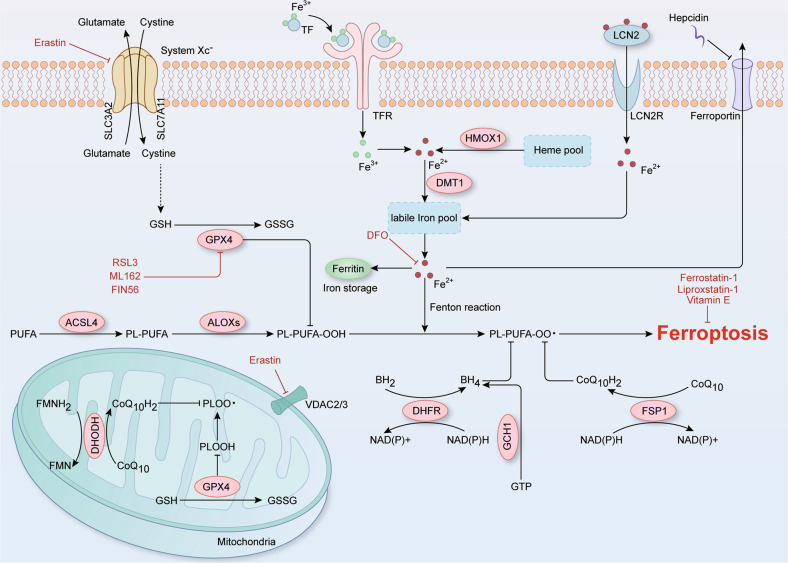
The mechanisms of ferroptosis. Iron-mediated lipid peroxidation is the core process in ferroptosis. In brief, TF and LCN2 carry extracellular iron and transfer it into cell via their corresponding receptors, while intracellular iron is mainly exported by ferroportin to maintain iron homeostasis. In the cells, HMOX1 degrades heme to release free iron, whereas the iron storage protein ferritin limits intracellular iron utilization to prevent iron overload and ferroptosis. As a catalyst, ferrous iron converts peroxides into free radicals such as hydroxyl and hydroperoxyl radicals, via the Fenton reaction, which causes excessive lipid peroxidation and ultimately triggers ferroptosis. During this process, the suppression of redox systems is an essential factor in ferroptosis. At present, four antioxidant pathways have been verified to be associated with ferroptosis: The system Xc^-^-GPX4, FSP1-CoQ_10_, GCH1-BH_4_, and DHODH-CoQ_10_ pathways. The antiporter System Xc^-^ containing SLC7A11 and SLC3A2 mediates the uptake of cystine, which is consumed during the synthesis of intracellular GSH. Next, the antioxidant enzyme GPX4 reduces lipid hydroperoxides into lipid alcohols via GSH to protect cells from ferroptosis. Thus, the inhibition of system Xc^-^-GPX4 by erastin, RSL3, ML162 or FIN56 induces ferroptosis, and therefore, these compounds have been applied to tumor therapy in animal experiments. Furthermore, FSP1, GCH1, DHFR, and DHODH can drive the production of antioxidants, such as BH_4_ and CoQ_10_H_2_ to defend against oxidative stress and ferroptotic cell death. Together, the joint dysregulation of iron metabolism and redox systems leads to the accumulation of intracellular lipid hydroperoxides, ultimately causing ferroptosis.

Abnormal redox system is another inducer of ferroptosis and multiple ferroptosis-related antioxidant signaling pathways have been verified, including the system Xc^-^-glutathione peroxidase 4 (GPX4), ferroptosis suppressor protein 1-coenzyme Q_10_ (FSP1-CoQ_10_), GTP cyclohydrolase 1-tetrahydrobiopterin (GCH1-BH_4_), and dihydroorotate dehydrogenase (DHODH)-CoQ_10_ pathways (Fig. 1) [17–19]. The aforementioned pathways are responsible for the synthesis of antioxidants, such as glutathione (GSH), CoQ_10_H_2_, and BH_4_, which decrease the level of intracellular oxidative stress and avoid ferroptosis [19–22]. In summary, the imbalance between the production of oxidants and antioxidant levels due to an abnormal redox system can lead to the accumulation of lipid peroxides and trigger ferroptosis.

**Fig. 2:**
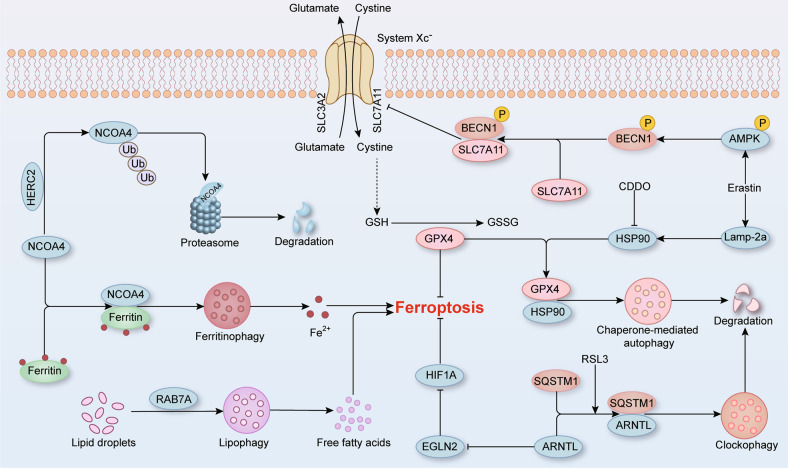
Ferroptosis is a type of autophagy-dependent cell death. NCOA4-mediated ferritinophagy induces ferritin degradation and iron overload, finally promoting oxidative injury and ferroptosis. E3 ubiquitin ligase HERC2 drives the proteasomal degradation of NCOA4 and enhances the stabilization of ferritin, which decreases intracellular levels of iron. RAB7A-mediated lipophagy and SQSTM1-mediated clockophagy both contributes to lipid peroxidation in ferroptosis. Erastin not only increases the expression of Lamp-2a, which promotes HSP90-mediated chaperone-mediated autophagy to degrade GPX4, but also promotes the combination of BECN1 and SLC7A11 to block System Xc- activity via AMPK phosphorylation.

Recently, researchers put forward the view that ferroptosis is a type of autophagy-dependent cell death (Fig. 2) [23, 24]. During ferroptosis, increased autophagy flux was observed and the expression of autophagy biomarker microtubule-associated protein 1 light chain 3 II (LC3 II) was significantly upregulated [25, 26]. Moreover, nuclear receptor coactivator 4 (NCOA4)-mediated ferritinophagy is the most representative manifestation of autophagy in ferroptosis [27, 28]. In the presence of iron-depletion, the cargo receptor NCOA4 binds to ferritin and delivers it to the autophagosome [27, 29]. Next, autophagosome combines with lysosomes to form autolysosome, which mediates the autophagic degradation of ferritin and subsequent iron release, called ferritinophagy [27]. Therefore, excessive activation of ferritinophagy causes intracellular iron overload, which further triggers oxidative stress and ferroptosis. In addition to iron metabolism, autophagy-mediated lipid peroxidation is another inducement for ferroptosis. In general, autophagy drives the digestion of lipid droplets, which products free fatty acids for mitochondrial beta-type oxidation, namely lipophagy [30]. However, activation of lipophagy leads to an excess of free fatty acids, which contributes to lipid peroxidation and thereby increases the propensity to ferroptosis [30]. The enhancement of lipid storage ability through increasing tumor protein D52 (TPD52) expression or the inhibition of lipophagy through silencing ATG5 and RAB7A can prevent RSL3-mediated lipid peroxidation and subsequent ferroptosis [30]. In addition, chaperone-mediated autophagy (CMA) and a novel selective autophagy clockophagy have been found to participate in ferroptosis via modulating the expression of aforementioned core molecules in redox system [31–35]. Together, these findings provide novel evidences for the fact that autophagy is indeed necessary for the process of ferroptosis.

So far, the mechanism and relevant findings have comprehensively been reviewed and interested readers can learn more information about ferroptosis through these review articles [3–5, 14, 17, 36–40].

## Signaling pathways in inflammation and their functions in ferroptosis

Inflammation is a highly protective mechanism against tissue injury, infection, and exposure to toxics/foreign particles and is the cumulative effect of a series of complex reactions involving multiple signaling pathways and molecules [41–44]. Studies have demonstrated that an abnormal inflammatory response is vital to iron metabolism disorder and redox system imbalance. Proinflammatory cytokines, such as IL-1β, IL-6, TNF-α, and IFN-γ, can regulate the synthesis of ferritin, which influences iron storage in cells and tissues [45]. Similarly, the activation of inflammation is accompanied by oxidative stress, which causes further redox system dysfunction and tissue injury. To expound the potential connection between ferroptosis and inflammation, we focus on multiple well-characterized inflammation-related signaling pathways, including the JAK-STAT, NF-κB, inflammasome, cGAS-STING and MAPK pathways, which are associated with inflammation, and discuss their roles in ferroptosis.

### JAK-STAT pathway in ferroptosis

**Fig. 3:**
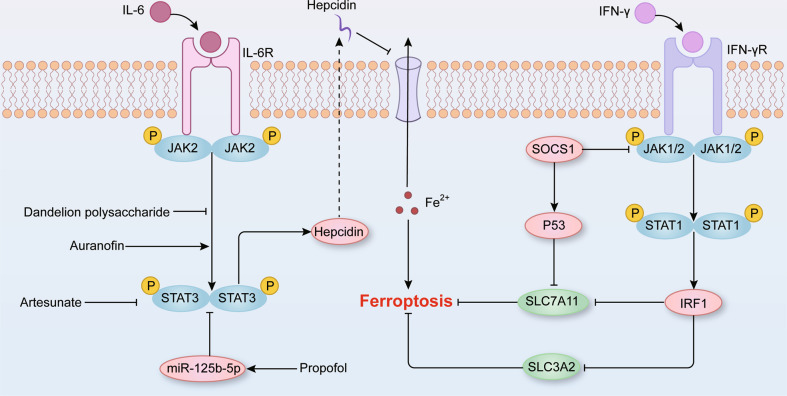
The role of JAK-STAT signaling pathway in ferroptosis. Cytokines, such as IL-6 and TNF bind to their corresponding receptors and thus induce the phosphorylation and activation of JAKs. In turn, activated JAKs phosphorylate STATs and induce their dimerization and nuclear translocation. Finally, STAT dimers bind to DNA sequences to induce the transcription of target genes and the activation of downstream signaling pathways. As a downstream target of the JAK-STAT pathway, phosphorylated STAT3 can increase hepcidin expression to inhibit iron export, resulting in ferroptosis. Moreover, activated STAT1 inhibits System Xc^-^ and exerts proferroptosis effects by via modulating IRF1.

The JAK-STAT pathway is a classical intracellular signal transduction pathway in multiple physiological and pathological processes (Fig. 3) [46, 47]. Several cytokines, such as ILs, IFNs, and granulocyte-macrophage colony stimulating factors, combine with corresponding transmembrane receptors and result in receptor oligomerization [48]. The close apposition of receptors promotes JAK phosphorylation and activation, which mediates the phosphorylation of STATs and thereby promotes the transcription of cytokine-responsive genes [48, 49]. In parallel, the activation of STAT boosts the expression of the suppressor of cytokine signaling (SOCS) family [50], which acts as the suppressors to establish a negative feedback mechanism [50].

IFN-γ is a crucial cytokine in antitumor host immunity and can increase the sensitivity of tumor cells to ferroptosis activators via the JAK -STAT signaling pathway [51, 52]. In erastin- or RSL3-induced ferroptosis of HCC cells, IFN-γ triggered the JAK-STAT-IRF1 signaling pathway and suppressed the transcription of system Xc^-^ subunits solute carrier family 3 member 2 (SLC3A2) and SLC7A11 [52]. Moreover, IFN-γ treatment alleviated the growth of xenograft tumors via increasing lipid oxidation in vivo [52]. Chromatin immunoprecipitation experiments confirmed that IFN-γ promoted the binding of STAT1 to SLC7A11 promoter and STAT1 deficiency reversed the contribution of IFN-γ to ferroptosis and lipid peroxidation [53]. In addition to tumorigenesis, IFN-γ hindered the synthesis of GSH via the JAK1-2-STAT1-SLC7A11 pathway and mediated retinal pigment epithelial cell ferroptosis, ultimately causing macular degeneration in vivo [54]. These findings suggest that the activation of the JAK-STAT signaling pathway by IFN-γ promotes ferroptosis, which might indicate a mechanism for immunotherapeutic management for oncotherapy.

As the second messenger of the JAK-STAT pathway, the STAT family participates in the regulation of ferroptosis-associated molecule expression, which further influences ferroptosis process. The widely used anesthetic propofol inhibited STAT3 expression via increased *miR-125b-5p* and accelerated gastric cancer cell ferroptosis [55]. Moreover, propofol decreased GPX4 and SLC7A11 protein levels by impairing STAT3 expression and slowed gastric cancer growth in vivo [55]. Similarly, propofol increased the intracellular iron content and ROS levels and exacerbated colorectal cancer cell ferroptosis by blocking STAT3 expression [56]. In addition to propofol, artesunate aggravated erastin-mediated ferroptosis by inhibiting STAT3 expression, exerting its antineoplastic effect on diffuse large B-cell lymphoma (DLBCL) in vitro [57]. As mentioned above, the SOCS family functions in a negative feedback loop in the JAK-STAT pathway to prevent sustained pathway activation and is also linked to ferroptosis [50]. SOCS1 overexpression drove p53 expression and increased ferroptosis sensitivity by reducing SLC7A11 expression and the GSH level in U2OS cells and IMR90 cells [58]. Moreover, treatment with peptidomimetic SOCS1 (MiS1) mitigated excessive tubular and vascular lipid peroxidation and thereby relieved inflammation and oxidative stress and ameliorated kidney lesions in black and tan brachyuric (BTBR) obese/obese mice [59].

Intracellular iron overload is a typical feature of ferroptosis and hepcidin profoundly regulates systemic iron homeostasis via its interaction with ferroportin [10, 60]. Interestingly, the IL-6-JAK2-STAT3 signaling pathway is well-characterized mechanism that drives hepcidin expression [60]. Recent studies reported that the anti-rheumatoid arthritis drug auranofin promoted hepcidin expression via canonical JAK2-STAT3 signaling pathway in both human liver cells and mice [61]. Similarly, dandelion polysaccharide reduced the expression of hepcidin by inhibiting JAK-STAT signaling pathway and thus affected iron burden and the process of hepatocellular carcinoma in a mouse model [62]. As mentioned above, iron overload contributes to the production of ROS through Fenton reaction and might cause cell injury and death [16]. A study found that propofol protected neuroblastoma cells SH-SY5Y against ferric citrate-induced oxidative stress and apoptosis by maintaining iron homeostasis [63]. Interestingly, these findings differed from those of another study suggesting that propofol increased the iron content and exacerbated the ferroptosis of cancer cells [55, 56]. With respect to this discrepancy, different metabolism modalities might reason for the different functions of propofol in different cell types. In general, metabolic reprogramming is a feature of cancer, and systemic iron metabolism is usually altered in cancer patients [64]. Thus, it is understandable that propofol displays adverse effects on iron metabolism in nontumor than in tumor cells. Furthermore, the different effects of propofol support its application for tumor therapy, in which normal cells would not become dysfunctional or injured because of iron overload. Nevertheless, the underlying mechanism of propofol function in iron metabolism needs to be clarified before clinical application.

As a central communication node, the JAK-STAT signaling pathway participates in the production of more than 50 cytokines and growth factors and is associated with various inflammatory diseases [46]. Recent studies have indicated that the JAK-STAT signaling pathway exerts large impacts on lipid peroxidation and ferroptosis by regulating redox systems and iron metabolism. In view of the complexity of the JAK-STAT signaling pathway, different stimuli might generate inconsistent and even adverse effects on ferroptosis, although these stimuli may similarly activate the signaling pathway. Consistent with these findings, some agents targeting the JAK-STAT signaling pathway have been proven to exert great therapeutic effects on ferroptosis related diseases, such as oncogenesis, in cell and animal models (Table 1). However, more work, including corresponding preclinical studies, is needed to explore the clinical transformation value of these agents. In addition, a number of JAK inhibitors, such as tofacitinib and peficitinib, have been applied to clinical treatments or assessed in clinical trials [47]. For example, ruxolitinib was the first JAK1/2 inhibitor approved by the FDA for use as a myelofibrosis therapy [65]. However, whether these specific inhibitors exert an impact on the ferroptosis process and can be applied to the treatment of ferroptosis-associated diseases is unclear, and more exploration and preclinical studies are needed.

**Table 1:** The functions of agents targeting inflammatory signaling pathways in ferroptosis-related diseases.

Inhibitors	Signaling pathways	Targets	Effects on ferroptosis	Functions	References
propofol	JAK-STAT	STAT3	exacerbates erastin-induced gastric cancer cell ferroptosis enhances iron content and ROS level, and induces colorectal cancer cell ferroptosis maintains iron homeostasis	represses gastric cancer growth in vivo inhibits colorectal cancer tumorigenesis protects against iron overload-induced nerve cell injury	[55, 56, 63]
artesunate	JAK-STAT	STAT3	enhances erastin-induced ferroptosis	exerts antineoplastic effects in DLBCL cells	[57]
dandelion polysaccharide	JAK-STAT	JAK2-STAT3	reduces the expression of hepcidin	decreases iron burden in tumor tissues	[62]
dimethyl fumarate	NF-κB	NF-κBp65	alleviates oxidative stress and ferroptosis	reduces chronic cerebral-induced neuronal damage and improves cognitive deficits	[72]
loganin	MAPK	ERK	attenuates cisplatin-induced renal tubular ferroptosis	decreases the severity of acute kidney injury	[121]
taxifolin	MAPK	p38 MAPK	reduces liver iron content and enhances redox status	mitigates iron overload-induced hepatocellular injury	[123]
alpha lipoic acid	MAPK	p38 MAPK	decreases MDA level and enhances SOD activity	ameliorates iron sucrose-induced oxidative kidney injury	[124]
resveratrol	MAPK	p38 MAPK	decreases lipid peroxide production and increases antioxidant activities	protects the spinal cord from ischemia injury	[125]
GDC-0879	MAPK	ERK1/2	upregulates GPX4 expression and prevents lipid peroxidation	ameliorates CoQ-deficiency kidney disease	[127]

*JAK* Janus kinase, *STAT* Signal transducer and activator of transcription, *ROS* Reactive oxygen species, *DLBCL* Diffuse large B cell lymphoma, *NF-κB* Nuclear factor-κB, *MAPK* Mitogen-activated protein kinase, *ERK* Extracellular signal-regulated kinase, *MDA* Malonaldehyde, *SOD* Superoxide dismutase, *GPX4* Glutathione peroxidase 4, *CoQ* Coenzyme Q.

### NF-κB pathway in ferroptosis

**Fig. 4:**
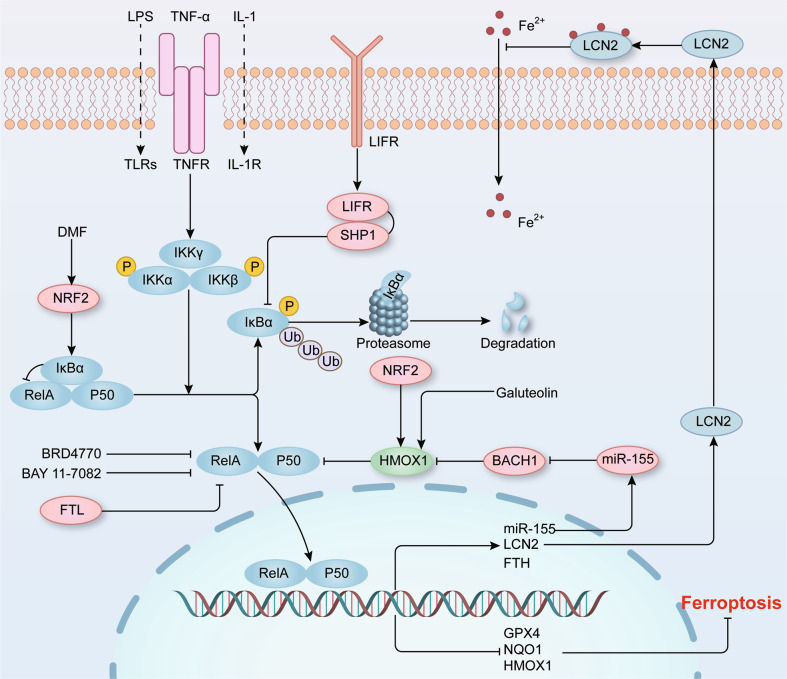
The role of NF-κB signaling pathway in ferroptosis. The classical signaling pathway is activated by Toll‑like receptor ligands (such as LPS), TNF, IL-1, and other stimuli. Under resting conditions, IκBα combines with NF-κB dimers to sequester NF-κB activity. These signaling molecules combine with their corresponding receptors and mediate the phosphorylation and subsequent degradation of IκBα. Then, the liberated NF-κB dimers are translocated to the nucleus and regulate target gene transcription. On the one hand, NF-κB can reduce the transcription of antioxidant molecules, such as GPX4, NQO1, and HMOX1, indicating the contribution of the NF-κB pathway to oxidative stress. On the other hand, the loss of LIFR enhances IκBα ubiquitination degradation and positively regulates NF-κB activation, which further promotes LCN2 secretion to sequester extracellular iron. In addition, a few agents, including BRD4770 and BAY 11-7082, exert their anti-ferroptosis effects via inhibiting NF-κB pathway.

NF-κB is a classical transcription factor that was discovered more than 30 years ago and plays an essential role in inflammation and innate immunity (Fig. 4) [66–69]. Canonical and noncanonical NF-κB pathways are two major signaling pathways that are activated through different mechanisms [69]. In canonical pathway, NF-κB p50/p65 heterodimers are translocated to the nucleus to mediate downstream gene transcription, whereas NF‑κB p52/RELB heterodimers are the core transcription factor of noncanonical pathway to modulate transcriptional activity [70]. It has been widely confirmed that NF-κB signaling pathway is complex and critical regulatory mechanism and participates in the regulation of inflammation and the immune system.

At present, many studies have revealed that ferroptosis involves the NF-κB signaling pathway. The deletion of leukemia inhibitory factor receptor (LIFR) enhanced the ferroptosis resistance of hepatocytes in vitro and is beneficial to liver tumorigenesis in vivo [71]. The loss of LIFR promoted the interaction of SHP1 and TRAF6, which positively regulated K63-linked ubiquitination and NF-κB signaling activation [71]. Furthermore, phosphorylated p65 promoted the transcription of LCN2, which hindered the import of extracellular iron and reduced hepatic cell sensitivity to ferroptosis inducers [71]. Similarly, dimethyl fumarate (DMF) alleviated neuroinflammation and ferroptosis by mediating NF-κB signaling pathway and improved two-vessel occlusion-induced cognitive impairment in a rat chronic cerebral hypoperfusion model [72]. DMF acted as the activator of NF-E2-related factor 2 (NRF2) and led to the upregulation of IκBα and inhibition of the NF-κB signaling pathway activation, promoting the expression of the crucial ferroptosis factors heme oxygenase 1 (HMOX1), NADPH quinone oxidoreductase 1 (NQO1) and GPX4, which ultimately protected cells against oxidative stress and ferroptosis [72]. In addition to the canonical pathway, noncanonical NF-κB activation has also been linked to ferroptosis. Similar to the ferroptosis inhibitor ferrostatin-1, hepatocyte-specific ablation of NIK or IKKα prevented excessive lipid peroxidation in primary hepatocytes and thus relieved APAP-mediated hepatotoxicity and mortality in liver tissue of mice [73]. In summary, targeting the NF‑κB signaling pathway is a promising approach for intervening in ferroptosis, leading to therapeutic effects on various diseases.

To date, certain approaches have been applied to establish ferroptosis model, in which it exists the activation of the NF-κB signaling pathway. RSL3 is a well- characterized ferroptosis inhibitor that inhibits the activity of GPX4 and thus leads to the excessive lipid peroxidation [22]. In RSL3-induced glioblastoma cell ferroptosis, the NF-κB signaling pathway was activated, which caused the transcriptional inhibition of ATF4 and xCT, resulting in excessive lipid peroxidation [74]. Furthermore, BAY 11-7082, an inhibitor of NF-κB signaling pathway activation, partially mitigated the antineoplastic function of RSL3 in vivo [74]. Similarly, our recent study revealed that in cystine deprivation-, imidazole ketone erastin- or RSL3-induced ferroptosis of smooth muscle cells (SMCs), the phosphorylation levels of NF-κB p65 were significantly increased, and histone methyltransferase inhibitor BRD4770 reversed the activation of the NF-κB signaling pathway induced by each of these three stimuli and protected SMCs against ferroptosis [8]. These results provide compelling evidence that the inflammatory response, especially the activation of the NF-κB signaling pathway, is essential for ferroptosis. Moreover, the classical ferroptosis inhibitor ferrostatin-1 mitigated LPS-mediated cardiac inflammation via the inhibition of TLR4-NF-κB signaling pathway in a rat cardiac dysfunction model and attenuated cardiac dysfunction, leading to reduced mortality [75]. Nevertheless, a few studies have reported different findings. Erastin is an inducer of ferroptosis and regulates the function of cystine-glutamate transport receptor, voltage-dependent anion channel (VDAC), and p53 [76]. A recent study reported that erastin alleviated LPS-mediated inflammatory cytokine production by inhibiting NF-κB activation in bone marrow-derived macrophages [77]. Moreover, erastin blocked the activation of the systemic inflammatory response and attenuated multiple organ damage in a CLP-induced sepsis model [77]. Hence, whether erastin-induced ferroptosis involves NF-κB signaling pathway activation remains to be further explored in the future.

HMOX1 catabolizes heme carbon monoxide, biliverdin, and ferrous iron, and thus is also an important ferroptosis regulation molecule [78]. In TNF-α-treated HUVECs, the NF-κB signaling pathway was activated, promoting the transcription of miR-155, which further bound to BACH1 mRNA and hindered its translation [79]. In the absence of BACH1, NRF2 combined with the HMOX1 antioxidant-response element and promoted HOMX1 expression [79]. Interestingly, another study reported that HMOX1 inhibited NF-κB RelA phosphorylation at serine 276 and, in turn, prevented acquisition of the proinflammatory phenotype, which reduced VCAM-1, ICAM-1, and E-selectin expression in TNF-stimulated ECs [80]. Additionally, galuteolin weakened TNF-α-induced IKKβ/NF-κB signaling pathway activation by increasing HMOX1 expression and mitigating the inflammatory response and proliferation in RA-FLS cells [81]. These results showed that in inflammatory diseases, the NF-κB signaling pathway components interact with HMOX1, but the roles played by these interactions remain to be clearly elucidated. In addition to HMOX1, other ferroptosis-related molecules are also regulated by the NF-κB signaling pathway. As an activator of TNFR, TNF-α elevated ferritin heavy chain 1 (FTH) expression via NF-κB signaling and inhibited apoptosis in serum-deprived hepatocellular carcinoma cells [82]. Moreover, the expression of the other element of ferritin, ferritin light chain (FTL), was increased in LPS-stimulated macrophages, which inhibited NF-κB signaling pathway activation and the production of TNF-α and IL-1β, subsequently alleviating inflammatory response in vitro and in vivo [83].

For a long time, NF-κB has been considered the central node in inflammation and its activation can further trigger a complex network that leads to corresponding responses depending on different stimuli [69]. As one mechanism in the innate immune response, the NF-κB signaling pathway serves as a mediator to participate in intracellular signal transduction and regulates proinflammatory gene expression, which ultimately prevents against bacterial and viral infections, cell damage and tumorigenesis [69]. The aforementioned studies showed the extensive influence of the NF-κB signaling pathway beyond its canonical functions to induce ferroptosis; however, further validation with clinical specimens is needed. Based on these findings, some agents exerted anti-ferroptosis effects and attenuate the adverse impacts of diseases by intervening in NF-κB signaling pathway activation in various cell and animal experiments. However, in contrast to those of JAK family, few specific inhibitors targeting the NF-κB signaling pathway have been applied in the clinic. Thus, the next step should focus on the exploration of specific inhibitors of this pathway.

### Inflammasome pathway in ferroptosis

**Fig. 5:**
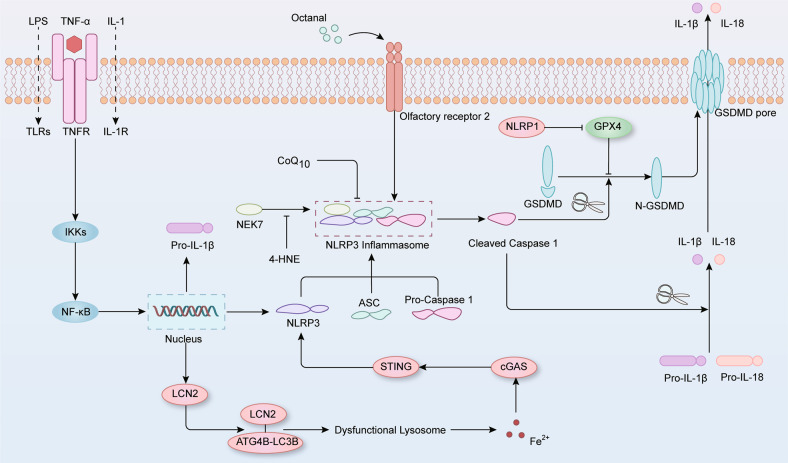
The role of inflammasome signaling pathway in ferroptosis. Membrane receptors sense inflammatory signals and, in turn, activate the NF-κB signaling pathway to induce NLRP3 and IL-1β transcription. NLRP3 recruits ASC and pro-caspase 1 to trigger the assembly of the NLRP3 inflammasome, which mediates the self-cleavage of pro-caspase 1. Activated caspase 1 further cleaves pro-IL-1β and pro-IL-18, which leads to the maturation of these proinflammatory cytokines. In parallel, gasdermin D is cleaved by caspase 1, and its N-terminal domain is transferred to plasma membrane, where it forms pores, mediating the release of mature IL-1β and IL-18 and causing cell lysis (pyroptosis). During this process, iron drives NLRP3 inflammasome formation via cGAS-STING pathway, whereas GPX4 blocks GSDMD cleavage to inhibit the inflammasome pathway. In addition, lipid peroxidation induced by octanal contributes to the production of the NLRP3 inflammasome, but 4-HNE binds to NLRP3 and thereby hinders its interaction with NEK7, suppressing inflammasome activation.

Inflammasomes are a group of macromolecular complexes that form in response to PAMPs and DAMPs (Fig. 5) [84]. Among inflammasomes, the NLRP3 inflammasome has been the most extensively studied and is involved in the process of innate immunity and autoinflammation [85]. NLRP3 senses the aforementioned stimuli and recruits the adaptor ASC and the effector pro-caspase 1 to assemble the NLRP3 inflammasome, resulting in the autoproteolytic activation of caspase 1 [86]. Activated caspase 1 further converts pro-IL-1β and pro-IL-18 to their mature forms, which are then secreted to drive a more potent inflammatory response [86]. Moreover, active caspase 1 cleaves the pore-forming protein GSDMD to release N-terminal GSDMD, which forms pores in the cytolemma to allow cytokine release and thus mediate pyroptosis [86].

As a well-studied multiprotein complex, the NLRP3 inflammasome has been shown to be possibly involved in ferroptosis. In a dry AMD-like mouse model, LCN2 was significantly increased in RPE cells, and the elevated LCN2 bound to ATG4B, obstructing autophagosome maturation [87]. Next, excessive intracellular ferrous iron accumulated, which drove the NLRP3 inflammasome activation via the cGAS-STING1 pathway and further induced oxidative stress, lipid peroxidation, and ferroptosis [87]. In MCT-induced pulmonary hypertension rats, the MDA content and labile iron pool were both increased, whereas the expression of GPX4 and FTH1 was suppressed in pulmonary artery endothelial cells [88]. Further investigation revealed that the protein expression of TLR4 and NLRP3 inflammasomes was high in monocrotaline-treated rats [88]. Moreover, ferrostatin-1 inhibited the activation of the TLR4-NLRP3 pathway and prevented ferroptosis‑mediated PAEC loss, delaying right ventricle hypertrophy and pulmonary vascular remodeling in vivo [88]. In addition to NLRP3, other inflammasomes are activated during ferroptosis. In H_2_O_2_-mediated HTR-8/SVneo cell ferroptosis, silencing of NLRP1 upregulated GPX4 expression and increased GSH levels, thereby alleviating intracellular MDA accumulation and cell death [89]. Moreover, ferroptosis inhibitor ferrostatin-1 alleviated the expression of NLRP1, NLRP3, IL-1β, and caspase-1, whereas the ferroptosis inducer erastin drove the activation of the NLRP1 and NLRP3 inflammasomes in placental trophoblast cells [89]. Taken together, these data suggest that the formation of inflammasomes might be a key step in ferroptosis.

Recently, a few studies have pointed out the possible connection between lipid peroxidation and inflammasome activation. For example, lipid peroxidation induced by octanal treatment increased LPS-induced IL-1β production, whereas the deletion of NLRP3 reversed the contribution of octanal to oxidative stress in macrophages [90]. Further studies verified that olfactory receptor 2 sensed the content of octanal in blood plasma and induced the activation of the NLRP3 inflammasome and secretion of IL-1β from macrophages, thereby driving the promotion of octanal on atherosclerosis in high-fat diet-induced mouse model [90]. In contrast, supplementation with CoQ_10_ enhanced mitochondrial function and decreased excessive lipid peroxidation, which ultimately impeded NLRP3 inflammasome formation and cell death in ETFDH-mutated lymphoblastoid cells [91]. Thus, the suppression of lipid peroxidation might be an appropriate mechanism to mitigate inflammasome activation and prolong cell survival. Interestingly, a few studies have shown inhibitory effects of lipid peroxidation on inflammasome formation. Hsu et al. found that the lipid peroxidation product 4-HNE directly bound to NLRP3 and blocked its interaction with NEK7, which ultimately inhibited the NLRP3 inflammasome activation and cell death induced by nigericin and ATP in human and mouse macrophages [92]. Moreover, the peritoneal administration of HNE or the GPX4 inhibitor RSL3, mitigated LPS-induced acute lung injury and sepsis by decreasing IL-1β and IL-18 secretion in vivo [92]. Therefore, the underlying mechanism of lipid peroxidation on NLRP3 remains unclear, and determination of whether lipid peroxidation promotes or inhibits inflammasome activation is worthy of further investigation.

As mentioned above, the GSDMD pore in the plasma membrane caused by inflammasome activation disrupts the osmotic potential, resulting in a cell death modality called pyroptosis [93]. Based on the interaction between ferroptosis and the inflammasome pathways, there might be a potential connection between ferroptosis and pyroptosis, as suggested by many studies. Immunohistochemical staining of human coronary artery specimens indicated that the pyroptosis-related protein NLRP3 was positively correlated with PTGS2 and ACSL4, which are essential components in ferroptosis execution [94]. Moreover, Kang et al. reported that GPX4 blocked GSDMD cleavage and served as a negative regulator of inflammasome activation [95]. In myeloid conditional GPX4-knockout mice, the lipid peroxidation rate was increased, which promoted GSDMD-dependent pyroptosis in macrophages via driving caspase-11 activation, leading to increased sepsis-induced lethality [95]. The application of vitamin E, the caspase inhibitor Z-VAD-FMK or wedelolactone reduced the serum levels of proinflammatory factors and reversed the contribution of GPX4 deficiency to lethal sepsis in vivo [95]. Similarly, the natural compound wedelolactone blocked pyroptosis and ferroptosis by increasing GPX4 expression and acted as an anti-inflammatory and antioxidant agent in a murine model of acute pancreatitis [96]. These findings provide new insights that diverse cell death modalities might interrelate with each other and maintain cellular homeostasis together.

### cGAS-STING pathway in ferroptosis

The cGAS-STING signaling pathway is an innate immune mechanism that can sense double-stranded DNA (dsDNA) to defend against extracellular or intracellular pathogens [97–99]. cGAS senses the cytosolic DNA that is produced by microbial infection or cell death, and then catalyzes the synthesis of the second messenger cGAMP [100]. Next, the ER membrane adaptor STING is oligomerized by binding with cGAMP and is then translocated from the ER to Golgi compartments, mediating TANK-binding kinase 1 (TBK1) autophosphorylation [100]. In turn, STING is phosphorylated by TBK1 and further recruits IFN regulatory factor 3 (IRF3) for phosphorylation by TBK1 [100]. Phosphorylated IRF3 undergoes dimerization and is translocated to the nucleus to mediate the transcription of type I IFNs and IFN-stimulated genes (ISGs), which participate in various pathophysiological processes, such as cardiac remodeling and ischemic stroke [100–107].

As the essential element in the type I IFN response, STING has been verified as a promoter of ferroptosis. Li et al. reported that the classical ferroptosis inducer erastin triggered mitochondrial oxidative stress and thereby increased the mitochondrial translocation of STING in human pancreatic cancer cells [108]. Furthermore, mitochondrial STING bound to MFN1/2 and promoted mitochondrial fusion, ultimately leading to mtROS production, lipid peroxidation, and ferroptosis in vitro [108]. Based on this finding, the knockdown of STING or MFN1/2 decreased cell sensitivity to ferroptosis and alleviate imidazole ketone erastin-mediated tumor suppression in a xenograft tumor model [108]. Similarly, the deletion of ICA69 decreased the expression of STING and thereby inhibited lipid peroxidation, ferroptosis, and the inflammatory response, which ultimately enhanced cardiac function and prolonged the survival rate after LPS-mediated septic cardiac dysfunction in vivo [109]. As a downstream target of STING, IRF3 participates in lipid peroxidation and ferroptosis. In Angiotensin II-induced cardiac hypertrophy mouse model, DHA protected against microvascular endothelial cell dysfunction and alleviated cardiac dysfunction by inhibiting ferroptosis [110]. During this process, the IRF3 level was increased by DHA and thus contributed to the transcription of SLC7A11, which further decreased the production of arachidonate 12-lipoxygenase and inhibited ferroptosis [110]. Collectively, these results demonstrate the effect of the activated cGAS-STING signaling pathway on lipid peroxidation and ferroptosis, and targeting this pathway may mitigate ferroptosis-mediated adverse effects.

In turn, ferroptosis can affect the cGAS-STING signaling pathway. Dai et al. reported that the high-iron diets or GPX4 deletion aggravated pancreatitis and promoted Kras-mediated pancreatic tumorigenesis by driving ferroptosis in mice [111]. Moreover, the increased oxidative stress caused by GPX4 depletion or a high-iron diet led to the release of the oxidative DNA damage product 8-OHG, which activated the TMEM173/cGAS dependent DNA sensor pathway and promoted macrophage infiltration in pancreatic tissues [111]. In contrast, another study reported that cellular lipid peroxidation induced by GPX4 inactivation alleviated the activation of the cGAS-STING signaling pathway. Increased lipid peroxidation promoted STING carbonylation, which hindered its transport from the ER to the Golgi complex and attenuated the activation of the STING DNA-sensing pathway in mouse peritoneal macrophage [112]. Furthermore, GPX4 deficiency or inhibition by RSL3 inhibited herpes simplex virus-1 (HSV-1)-induced innate antiviral immune responses and exacerbated HSV-1 replication in vivo, suggesting a destructive effect of lipid peroxidation in the innate immune response [112]. Hence, whether ferroptosis promotes or inhibits the cGAS-STING signaling pathway depends on the disease states, which should be further studied.

### MAPK signaling pathway in ferroptosis

The MAPK family is a group of serine/threonine protein kinases that can mediate a series of enzymatic reactions in a wide range of inflammatory responses [113, 114]. In brief, MAPK signaling pathways are capable of activating downstream effector kinases or regulate the function of transcription factors to generate inflammatory and immune responses [113, 115–118]. Notably, these MAPKs engage in crosstalk with each other and regulate cellular homeostasis together. For example, p38α-mediated MSK1/2 activation promotes the transcription of DUSP1, which in turn inhibits the activation JNK-JUN pathway [116]. Therefore, the MAPK signaling pathway is a complicated signal transduction mechanism and more attention should be given to not only the role it plays various biological processes, but also to its internal connections.

Similar to that of other pathways, the activation of MAPK pathway-dependent inflammation is involved in ferroptosis. In a neonatal rat hypoxia-ischemia model, the TLR4-p38 MAPK pathway was activated, which promoted the production of the proinflammatory cytokines IL-1β, IL-6, and IL-18 and simultaneously decreased the expression of SLC7A11 and GPX4, leading to neuroinflammation and ferroptosis [119]. Similarly, oxygen-glucose deprivation (OGD) triggered the TLR4-p38 MAPK pathway and then increased the accumulation of MDA, leading to neuronal cell ferroptosis, whereas the p38 inhibitor SB203580 alleviated OGD-induced ferroptosis by upregulating SLC7A11 and GPX4 expression in neuronal cells [119]. In addition to p38 MAPK, ERK is an intermediary that mediates the inflammatory response and ferroptosis. Exposure to cadmium telluride quantum dots (CdTe QDs) enhanced ferritinophagy by regulating the NRF2-ERK pathway and thereby caused iron release from the labile iron pool, resulting in macrophage ferroptosis and an inflammatory response [120]. Notably, some agents targeting the ERK pathway have been shown to exert ideal therapeutic effects that attenuate ferroptosis. In cisplatin-induced acute kidney injury, loganin attenuated renal cell ferroptosis and the release of the proinflammatory cytokines IL-1β, IL-6, and TNF-α via the inhibition of ERK activation in vivo [121]. In summary, the suppression of the MAPK signaling pathway might be an appropriate approach to intervene in ferroptosis-associated diseases, but more specific agents with this activity remain to be discovered.

As typical features of ferroptosis, intracellular iron overload and excessive lipid peroxidation accumulation have been confirmed to influence the activity of the MAPK pathway. In CTX-induced muscle injury, iron overload inhibited the phosphorylation of ERK1/2 and p38, and thereby increased oxidative stress and aggravated muscle regeneration [122]. In contrast, another study showed that excessive iron led to the upregulated phosphorylation of p38 and c-FOS and aggravated hepatocellular injury along with increasing lipid peroxidation in iron-treated rats [123]. The natural compound taxifolin reversed iron-mediated oxidative stress, enhanced the redox status and extended hepatocellular survival by inhibiting MAPK signaling pathway activation in vivo [123]. Recently, researchers found that a few agents exert their antioxidant effects via the MAPK signaling pathway. For example, the antioxidant alpha lipoic acid (ALA) enhanced the activity of SOD and decreased MDA content by suppressing the p38 MAPK signaling pathway in iron overload-induced kidney injury [124]. Similarly, another antioxidant, resveratrol, suppressed the iNOS-p38 MAPK pathway and prevented spinal cord ischemia/reperfusion injury-mediated lipid peroxidation accumulation in mice [125]. As previously mentioned, CoQ_10_ is a vital element that maintains redox homeostasis and inhibits ferroptosis [126]. The treatment with GDC-0879, a Braf/MAPK-targeting inhibitor, prevented PUFA-mediated lipid peroxidation and reversed CoQ deficiency, resulting in cell death and kidney disease in vivo [127]. Taken together, these studies suggest that further exploration of the link between the MAPK signaling pathway and lipid peroxidation will likely lead to discovery of targets for the intervention of ferroptosis-related diseases.

## Conclusions and perspectives

In this review, we summarized five classical inflammation-related signaling pathways, namely, JAK-STAT, NF-κB, inflammasome, cGAS-STING, and MAPK signaling pathways, and expounded on their roles in ferroptosis. As discussed previously, these pathways are involved in ferroptosis, and ferroptosis can regulate the activity of these pathways, affecting cellular biological function. In addition, targeting these pathways may enable effective intervention in ferroptosis and thereby exert therapeutic effects on various disease models in vivo (Table 1). Hence, we can conclude that ferroptosis and inflammation are closely linked and together realize physiological and pathological functions. Indeed, other cell death types, such as necroptosis and pyroptosis, are also associated with inflammation and related signaling pathways [128–131]. Even so, ferroptosis has unique connection with inflammation owing to its own mechanism, compared to other cell death forms (Table 2) [128–131]. In addition, many studies have reported that inflammatory-related pathways participate in the regulation of iron metabolism, lipid peroxidation, and redox systems, which has helped us better understand the mechanism of these pathways in ferroptosis. Based on the aforementioned findings, a few agents targeting inflammatory signaling pathways have been verified to exert effects on iron metabolism and lipid peroxidation and drive anti- or pro-ferroptosis therapeutic outcomes. Collectively, these studies on inflammatory signaling pathways provide novel insights into possible interventions in ferroptosis-related diseases. Nevertheless, recent studies have only reported this phenomenon that inflammatory pathways affect oxidative stress and ferroptosis, but the detailed molecular mechanisms are still unknown.

**Table 2:** Comparison between non-inflammatory cell death and proinflammatory cell death.

	Ferroptosis	Necroptosis	Pyroptosis	Apoptosis	Autophagic cell death
Morphology	Increased plasma and mitochondrial membrane density, mitochondrial crest fracture, disappeared mitochondrial cristae	Organelle swelling, cell membrane rupture, degradation of cytoplasm and nucleus	Cell swelling, membrane blistering, DNA breakage, cell membrane rupture	Cell shrinkage, chromatin condensation, membrane blebbing, DNA fragmentation	Golgi and endoplasmic reticulum swelling, broken nucleus, the formation of a large number of phagocytic vesicles
Features	Intracellular iron overload, excessive lipid peroxidation	Necrosome complex formation, caspase-8 deficiency	Inflammasome formation, GSDMD pores on plasma membrane, IL-1β and IL-18 production and excretion	Apoptotic body formation, caspase family activation	Autophagic body formation, ATG proteins activation
Molecular biomarkers	HMOX1, PTGS2, GPX4, SLC7A11, Ferritin, FSP1, TFR1	p-RIP1, p-RIP3, p-MLKL	NLRP3, GSDMD, Caspase-1, IL-1β	Caspase-3, BCL2, BAX, P53	P62, LC3 II, ATG5, ATG7, Beclin-1
Inducers	Erastin, RSL3, FIN56	RIP1/RIP3/MLKL activator 1	Lipopolysaccharide	Actinomycin, camptothecin, cyclotleximide	Brefeldin A, carbamazepine rapamycin
Inhibitors	Ferrostatin-1, liprostatin-1, deferoxamine	Necrostatin-1	VX765, Z-VAD-FMK	Z-VAD-FMK	3-Methyladenine, bafilomycin A1, hydroxychloroquine
Measurements	BODIPY 581/591 C11 assay, lipid peroxidation (MDA) assay, iron content assay, GSH and GSSG assay	Annexin V/PI assay, mitochondrial membrane potential assay	ELISA assay (IL-1β and IL-18), N-GSDMD and cleaved IL-1β content, caspase activation	Annexin V/PI assay, mitochondrial membrane potential assay, caspase activation	Autophagic flux assay, mCherry-eGFP-LC3 II fluorescence staining
Inflammation	Proinflammatory	Proinflammatory	Proinflammatory	Non-inflammatory	Non-inflammatory
major molecules related to inflammation	SLC7A11, GPX4, FSP1	RIP1, RIP3, MLKL	NLRP3, Caspase-1, GSDMD	Caspase-3, Caspase-8, Caspase-9	LC3 II, ATG5, ATG7
Major inflammatory signaling pathways	JAK-STAT, NF-κB, inflammasome, cGAS-STING and MAPK signaling pathways	JAK-STAT, NF-κB, inflammasome, cGAS-STING and MAPK signaling pathways	JAK-STAT, NF-κB, inflammasome, cGAS-STING and MAPK signaling pathways	JAK-STAT, NF-κB, inflammasome, cGAS-STING and MAPK signaling pathways	JAK-STAT, NF-κB, inflammasome, cGAS-STING and MAPK signaling pathways

*GSDMD* Gasdermin D, *IL-1β* Interleukin-1β, *ATG* Autophagy-related gene, *HMOX1* Heme oxygenase 1, *PTGS2* Prostaglandin endoperoxidase synthase 2, *GPX4* Glutathione peroxidase 4, *SLC7A11* Solute carrier family 7 member 11, *FSP1* Ferroptosis suppressor protein 1, *TFR1* Transferrin receptor 1, *p-RIP1* Phosphorylated receptor interacting protein kinase 1, *MLKL* Mixed lineage kinase domain-like protein, *NLRP3* NOD-like receptor family pyrin domain containing 3, *BCL2* B-cell lymphoma 2, *LC3 II* Microtubule-associated protein 1 light chain 3 II, *MDA* Malonaldehyde, *GSH* Glutathione, *GSSG* Glutathione persulfide.

As an essential trace element, iron is necessary to maintain the biological functions of cells, but its overload causes intracellular oxidative stress and ferroptosis [16]. Systemic iron homeostasis is a complicated physiological process, in which various cells and molecules participate [16]. As the core node that modulates the distribution of iron in different organs, hepcidin is synthesized and secreted by hepatocytes and can hinder intracellular iron export by degrading ferroportin [132, 133]. Recent work has confirmed that the inflammatory response, especially the involvement of the IL-6/JAK/STAT3 signaling pathway, is the critical regulatory mechanism for hepcidin expression [60, 132]. Therefore, inflammation is a vital link for systemic iron homeostasis and inflammatory signaling pathways might be bridges to help maintain the iron balance in different organs. Moreover, iron metabolism may connect the inflammation to ferroptosis. However, there is not enough evidence to confirm these suppositions, and more exploration into this possible connection is a worthy research direction.

Upon inflammatory stimulation, these signaling pathways interact with each other and cooperatively modulate intracellular signal transduction. After cGAS-STING signaling pathway activation, phosphorylated TBK1 mediates the activation of NF-κB, promoting the transcription of IL-6 and TNF to drive the innate immune response [134]. Similarly, activated NF-κB promotes the transcription of pro-IL-1β and NLRP3 and thus regulates the formation of inflammasomes [135]. Although ferroptosis is a complex biological process that is regulated by multiple inflammatory signaling pathways, studies have consistently focused on one pathway, not comprehensive analyses of these pathways together. Whether the connections among the aforementioned pathways are unchanged in ferroptosis remains to be studied. Fortunately, a few studies have indicated a possible link among these pathways in ferroptosis. As mentioned above, low doses of auranofin caused intracellular iron overload by upregulating hepcidin expression via the NF-κB/IL-6/JAK-STAT signaling pathway [61]. Moreover, Schmitt et al. reported that dimethyl fumarate simultaneously mediated the succination of the kinases IKK2 and JAK1 and inhibited NF-κB and JAK-STAT3 signaling pathways, which caused DLBCL cell ferroptosis [136]. However, more direct and precise evidence needs to be provided to verify the connection of these pathways in ferroptosis.

To date, based on the roles of inflammation in ferroptosis, many agents targeting the aforementioned pathways have exerted great therapeutic effects in ferroptosis-associated diseases, such as tumorigenesis and ischemia/reperfusion injury. The next steps might involve further analysis of the pharmacological mechanisms of these agents and the exploration of more convincing evidence to clarify their clinical transformation value. Remarkably, a few novel drugs targeting these pathways, such as tofacitinib and ruxolitinib, have been applied to clinical therapeutics. Whether these drugs can exert therapeutic effects on ferroptosis-associated diseases remains to be clarified, and corresponding studies need to be done to expand the application of these drugs. Of course, some issues need to be considered when carrying out relevant studies. Inflammation is an essential biological process in response to exogenous and endogenous danger signals; thus, maintaining the balance between the regulation of ferroptosis via inflammatory signaling pathways and the stabilization of the immune system through the whole body is a complex challenge. In addition, inflammatory signaling pathways are intricate networks that lead to mutual effects, and targeting one site and preventing unanticipated and adverse cascade reactions are problems to be resolved. Nevertheless, we insist that focusing on inflammation, especially the classical multi-potent signaling pathways, is significantly important for the study of ferroptosis and will provide novel and meaningful discoveries for the treatment of associated diseases.

## Data Availability

All data analyzed during this study are included in this published article. All data are available from the corresponding author on reasonable request.
